# The potential role of human endogenous retrovirus K in glioblastoma

**DOI:** 10.1172/JCI170885

**Published:** 2023-07-03

**Authors:** Parvinder Hothi, Charles Cobbs

**Affiliations:** Ivy Center for Advanced Brain Tumor Treatment, Swedish Neuroscience Institute, Seattle, Washington, USA.

## Abstract

The most active human endogenous retrovirus K (HERV-K) subtype, HML-2, has been implicated as a driver of oncogenesis in several cancers. However, the presence and function of HML-2 in malignant gliomas has remained unclear. In this issue of the *JCI*, Shah and colleagues demonstrate HML-2 overexpression in glioblastoma (GBM) and its role in maintaining the cancer stem cell phenotype. Given that stem-like cells are considered responsible for GBM heterogeneity and treatment resistance, targeting the stem cell niche may reduce tumor recurrence and improve clinical outcomes. The findings provide a foundation for future studies to determine whether antiretroviral and/or immunotherapy approaches targeting HML-2 could be used as therapeutics for GBM.

## Glioblastoma pathogenesis and therapeutic approaches

Glioblastoma (GBM) is the most common primary brain tumor in adults, with approximately 10,000 new cases every year in the USA. With surgery, radiation, and chemotherapy, the median survival is approximately 15 months after diagnosis, and it has remained relatively unchanged over the last several decades. Despite intensive research, only a few rare germline genetic mutations and rare environmental exposures are known to be associated with risk of GBM (1). Current evidence indicates that GBMs likely arise from resident glial precursor stem cells in the subventricular zone of the brain. Acquired DNA mutations in these stem cells can promote development of an immortalized glial precursor stem-like cell population that evolves into daughter GBM cells with the capacity to invade and destroy the brain (2). Key properties of these GBM stem-like cells include the expression of genes that promote the stem-like state (e.g., *SOX2*, *OCT4*) and facilitate tumor proliferation and resistance to radiation and chemotherapy (3). In addition to the highly proliferative and invasive properties of GBMs, these tumors are also cloaked in an immunosuppressive tumor microenvironment and are therefore termed “cold” tumors (4). As a result, immune checkpoint blockade (ICB) immunotherapy approaches to GBM have shown very limited success, and different targets of immunotherapy are desperately needed for this disease (5). Thus, two major questions regarding our understanding of GBM pathogenesis and therapy remain: (a) What factors contribute to the early events in glial stem cell oncogenesis, and (b) are there GBM tumor-associated antigens that could synergize with ICB to improve immunotherapy?

**Figure 1:**
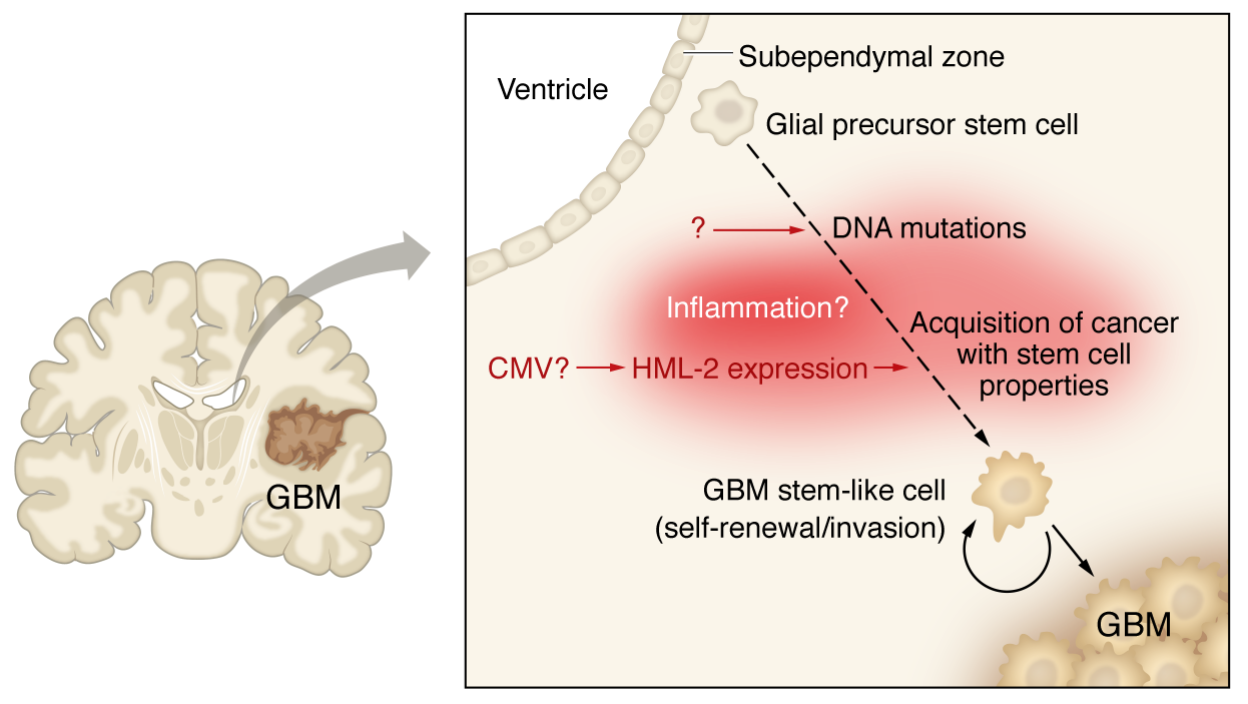
HML-2 drives the GBM cancer stem cell phenotype and promotes GBM pathogenesis. GBM stem-like cells are thought to derive from subependymal zone glial precursor stem cells. Mutations in these glial precursor cells contribute to transformation and development of GBM stem-like cells, which often express OCT4, SOX2, etc. These cells promote further GBM cell proliferation and invasion and resistance to chemotherapy. Shah and colleagues showed that HML-2 drives the GBM cancer stem cell phenotype and, thus, promotes GBM pathogenesis (6).

It is in the context of these two knowledge gaps that the findings by Shah et al. potentially point the field in another direction (6). In this issue of *JCI*, Shah and colleagues report that a human endogenous retrovirus (HERV) might be contributing to GBM pathogenesis (Figure 1) and might also serve as a target for immunotherapy (6).

## HML-2 promotes an aggressive glioblastoma phenotype

Using sophisticated methodologies, including single-cell RNA-Seq, Shah et al. identified the expression of HML-2, a subtype of the human endogenous retrovirus K (HERV-K) family, in human GBM. About 8% of the human genome is composed of bits and pieces of integrated retroviral genomes that have become spliced into the mammalian genome over the millennia. Most of these HERV genomes are degenerated and no longer encode for proteins or competent retroviral particles. However, the most recently acquired HERV-K, HML-2, has many integration sites in the human genome and can still encode for functional retroviral proteins, but it is normally transcriptionally silenced. Indeed, HML-2 is capable of expressing intact, retroviral particles and proteins, including GAG, LYR5Hs, POL, and ENV. Normally, expression of HERV-K is found to occur only during human embryogenesis, but increasing evidence indicates HERV-K genome and proteins may occur in association with human malignancies and even participate in oncogenesis (7).

Shah and colleagues explored human GBM for evidence of HML-2. They used multiplex immunofluorescence and, in a survey of nine GBMs, identified evidence of HML-2 ENV protein in eight of nine tumors (89%). HML-2 ENV protein was not found in normal epilepsy-derived brain tissue. Transcriptome analysis identified several HML-2 transcripts that were more highly associated with GBM compared with healthy brain. These HML-2 transcripts were also more highly expressed in aggressive phenotypes of GBM. Furthermore, single-cell RNA-Seq analysis of 11 GBM tumors showed increased expression of HML-2 in the GBM precursor stem-like cells. Indeed, the HML-2 *LTR5Hs* gene, which has functionality as a transcription factor binding site and genomic enhancer for downstream genes and can activate the *OCT4* stem cell gene, was found in its full-length functional state in six human chromosomes that were overexpressed in stem-like GBM cells compared with more differentiated GBM tumor cells. Consistent with these findings, the authors found that HML-2 expression was located in the same tumor cells in GBM as those that expressed critical markers of tumor stem cell biology, such as OCT4, Nestin, and SOX-2 (6).

To determine whether HML-2 expression was functionally relevant in the GBM cells, the authors depleted HML-2 using CRISPR interference. This strategy demonstrated that downregulation of HML-2 resulted in decreased viability of neurosphere formation, which is a hallmark of glioma stem-like cells. Conversely, when HML-2 sequences were added into normal human glial stem cells, these cells had increased expression of stem cell–like gene *OCT4*, and they were more invasive. In mouse GBM models, forced expression of HML-2 ENV in human GBM xenograft cells promoted tumor growth while knockdown diminished tumor growth and improved mouse survival (2).

Further proof of principle was shown when antiretroviral nucleoside reverse transcriptase inhibitor abacavir could diminish the cell viability of patient-derived GBM neurospheres. This finding was associated with decreased expression of the HML-2 ENV gene and cellular OCT4 protein.

## Conclusions and implications

Shah et al. (6) provide substantial evidence that HML-2 is transcriptionally active and expressing retroviral gene products and proteins in human GBM tumor cells. These HML-2 gene products are correlated with GBM stem cell factors such as OCT4, and upregulation and downregulation of HML-2 expression in GBM neurospheres and xenografts can increase and decrease the GBM stem cell properties, respectively.

Over 20 years ago, a similar story emerged with CMV that underscores the importance for understanding the role(s) of viral expression in GBM yet exemplifies the elusive nature of such investigations. In 2002, we found that CMV was associated with GBM (8). Despite two decades of further research, its role continues to be controversial. We and others demonstrated that CMV was present and expressed in GBM stem cells, could drive stem cell biology (9, 10), and could contribute to GBM immune suppression (11). CMV-based vaccine approaches aimed at GBM have also shown promise (12). However, efforts to sequence CMV genomes from GBM have remained problematic, hindering the advancement of the CMV-GBM story. A recent report that demonstrates isolation of potentially oncogenic GBM-associated CMV variants may renew interest in this area (13).

The implications of Shah et al. (6) are potentially vast. Corroboration of the findings could open an entirely different understanding of the etiology and therapeutics in GBM. Many questions immediately come to mind. First, is HML-2 a fundamental contributor to human GBM oncogenesis? It is known that HML-2 can influence cancer biology and has been associated with other malignancies (14). Follow-up questions include the following. What might reactivate HML-2, which is normally transcriptionally repressed by DNA methylation, in normal glial precursor cells? Does HML-2 itself transform glial cells or just promote the stem cell phenotype? Are there cofactors that may contribute to HML-2 activation? There is evidence that neuroinflammation, and other pathological agents like CMV, could induce expression of dormant HML-2 (15, 16).

Additionally, are there therapeutic implications with respect to the identification of HML-2 expression? Can antiretroviral reverse transcriptase inhibitors be utilized to reduce the HML-2 expression and the tumor-promoting effects of HML-2 on GBM pathogenesis? The data presented in Shah et al. indicate that this may be possible (6). Finally, could HML-2 antigens (e.g, GAG, POL, ENV, LTR5Hs) that are expressed in GBM cells, but not in normal brain, be targeted by immunotherapeutic approaches, perhaps in combination with ICB? This is an intriguing question, and some preliminary evidence suggests this approach is feasible (17).

## Version 1 07/03/2023

Electronic publication
